# Use of Acellular Umbilical Cord-Derived Tissues in Corneal and Ocular Surface Diseases

**DOI:** 10.3390/medicines8020012

**Published:** 2021-02-09

**Authors:** Arianna A. Tovar, Ian A. White, Alfonso L. Sabater

**Affiliations:** 1 Department of Ophthalmology, Bascom Palmer Eye Institute, University of Miami Miller School of Medicine, Miami, FL 33136, USA; aat128@miami.edu; 2 Neobiosis, LLC, UF Innovate Biotechnology Institute, 12085 Research Drive, Alachua, FL 32615, USA

**Keywords:** blood derived products, ophthalmology, ocular surface, umbilical cord blood, umbilical cord serum, extracellular vesicles, tissue regeneration

## Abstract

Blood derived products have become a valuable source of tissue for the treatment of ocular surface diseases that are refractory to conventional treatments. These can be obtained from autologous or allogeneic sources (patient’s own blood or from healthy adult donors/umbilical cord blood, respectively). Allogeneic cord blood demonstrates practical advantages over alternatives and these advantages will be discussed herein. Umbilical cord blood (UCB) can be divided, generally speaking, into two distinct products: first, mononuclear cells, which can be used in regenerative ophthalmology, and second, the plasma/serum (an acellular fraction), which may be used in the form of eyedrops administered directly to the damaged ocular surface. The rationale for using umbilical cord serum (UCS) to treat ocular surface diseases such as severe dry eye syndrome (DES), persistent epithelial defects (PED), recurrent epithelial erosions, ocular chemical burns, graft versus host disease (GVHD), among others, is the considerably high concentration of growth factors and cytokines, mimicking the natural healing properties of human tears. Allogeneic serum also offers the opportunity for therapeutic treatment to patients who, due to poor heath, cannot provide autologous serum. The mechanism of action involves the stimulation of endogenous cellular proliferation, differentiation and maturation, which is highly efficient in promoting and enhancing corneal epithelial healing where other therapies have previously failed.

## 1. History of Blood Derived Products in Ophthalmology

The idea of using blood derived products in ophthalmology has become increasingly popular in the past few decades. Starting in 1975, when Ralph et al. created a mobile ocular perfusion pump for continuous delivery of autologous serum (AS) to treat various ocular surface diseases such as keratoconjunctivitis sicca (KCS), persistent epithelial defects, Stevens Johnson syndrome, ocular pemphigoid, chemical burns, or following penetrating keratoplasty [1]. In 1984 Fox, et al. treated KCS patients with the first generation of such blood derived products, specifically AS tears [2]. Later, other than the patient’s own peripheral blood (autologous), additional sources were used to obtain serum products from healthy donors (allogeneic). These sources include allogeneic adult donor’s blood and umbilical cord blood (UCB).

## 2. Ocular Surface and Normal Tear Film

The cornea, responsible for most of the overall refractive power of the eye, is a transparent, avascular tissue that serves as a mechanical and functional barrier for the delicate intraocular structures of the eye. It receives its nutrients and oxygen by simple and facilitated diffusion, posteriorly from the aqueous humor and anteriorly from the tear film. Normal tear film consists of water, lipids and mucins, along with a variety of cytokines, Interleukins (ILs) and growth factors (GFs), such as epidermal growth factor (EGF), fibroblast growth factor (FGF), platelet-derived growth factor (PDGF), keratinocyte growth factor (KGF), transforming growth factor (TGF), nerve growth factor (NGF) insulin-like growth factor (IGF), Vitamin A, among others. Each of these play an important role in the physiological ocular surface cellular turnover, the maintenance of transparency of the cornea and surface wound healing [3,4]. Both peripheral blood serum (PBS, either autologous or allogeneic) and UCB serum (UCS) are currently used as artificial “tears” to treat ocular surface diseases because they contain high concentrations of the above cytokines and GFs. Their mechanism of action involves the stimulation of corneal and conjunctival epithelial cellular proliferation, differentiation and maturation, which mimics the normal function of natural tears [5]. This offers an advantage over other conventional ocular surface therapies, such as pharmacological drugs, which simply lubricate the eye and are not replete with epitheliotropic growth factors [3].

Among the different sources of blood-derived products, autologous serum is the most commonly known and most widely used. However, the use of allogeneic products, specially UCB, has increased recently in the ophthalmologic field. First, the “off-the-shelf” ease of use facilitates rapid outpatient procedures. Second, it offers therapeutic opportunities for patients with systemic diseases (those whose inflammatory mediators, within their own serum), blood dyscrasias, anemia or other conditions where the extraction of peripheral blood might be impractical. Third, UCB can be fractioned into two distinct products, which broaden its therapeutic range. With minimal manipulation whole UCB can be rendered into a mononuclear cellular product rich in hematopoietic stem (HSCs) and progenitor cells (HPCs), mesenchymal stromal cells (MSCs), epithelial cells and endothelial progenitor cells (EPCs), potent mediators of repair for regenerative retinal or corneal medicine. With appropriate isolation an acellular product can also be extracted, which can be used as eye drops to be applied topically to treat ocular surface diseases such as dry eye syndrome or persistent corneal epithelial defects of multiple etiologies, when other conventional therapies have failed [6,7].

## 3. Preparation

**Figure 1:**
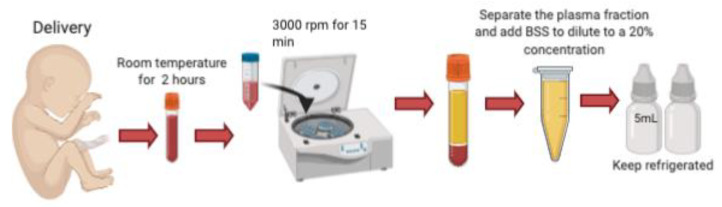
Preparation of umbilical cord serum tears. UCB is collected from the umbilical vein following delivery. It is kept at room temperature without anticoagulants for 2 h to induce clotting. It is centrifugated and the serum separated under sterile conditions. The serum is diluted with sterile saline to a 20% concentration, and aliquoted into sterile 5 mL droppers.

Umbilical cord blood is obtained immediately following cesarian delivery. Informed consent is obtained from the mother, who must undergo screening for HIV, Hepatitis B, Hepatitis C, and other sexually transmitted infections including CMV, Toxoplasma, Syphilis and HTLV twice, the first one at 8 and then at 38 gestational weeks, to prevent the transmission of these blood-borne diseases. A volume of 200–250 microliters [3] to 60–80 mL [5] of umbilical cord blood is collected from the umbilical vein after fetal delivery, and it must be allowed to clot by keeping it for 2 h at room temperature without anticoagulant. It is then centrifugated at 3000 r.p.m for 15 min and the serum is carefully separated under sterile conditions. The serum is diluted with sterile saline (NaCl) to a 20% concentration, and aliquoted into sterile 5 mL bottles using sterile technique. Full bottles must be stored in the freezer at −20 °C for a maximum of 6 months prior to use. Because UCS contains antibacterial agents such as lysozyme, IgG and complement factors, each having bacteriostatic properties, these products can be manufactured preservative-free, which dramatically reduces the risk of toxic reactions on the ocular surface [3,5,8] (Figure 1).

## 4. Growth Factors and Interleukin Content

In a 2018 study conducted in Bologna, Italy [8], the relative concentrations of growth factors (GFs) and interleukins (ILs) found in the peripheral blood serum (PBS) and UCS of pregnant women was investigated. The authors measured levels of EGF, FGF, PDGF, IGF, TGF-α, TGF-β 1-2-3, VEGF, NGF, IL-1β, IL-4, IL-6, IL-10 and IL-13, and found that EGF, TGF-α, TGF-β2, FGF, PDGF, VEGF, NGF, IL-1B, IL-4, IL-6, IL-10, and IL-13 were expressed at significantly higher levels in UCS compared to PBS, while the levels of IGF-1, IGF-2, and TGF-β1 were significantly higher in PBS compared to UCS (Table 1). They also reported that the female sex of the developing fetus was positively correlated with the concentrations of EGF and PDGF levels in UCS, and that weight was negatively correlated with EGF, specifically, that the more weight, the lower the EGF concentration [8]. These results were consistent with a previous study [9], which did not assay for such a large panel of GFs and IL, yet still showed that EGF and TGF- β concentrations were of greater abundance in UCS compared to PBS. Conversely, Vitamin A concentration was found to be greater in PBS compared to UCS [9]. Corneal healing is complex and involves migration, proliferation and differentiation of cells, and each are mediated by GFs and ILs. This occurs only when their concentration is within an appropriate range, as excessive concentrations can lead to extracellular matrix scars or stromal haze [8,10]. For example, normal tear concentration of EGF ranges between 0.7–9.7 ng/mL [11,12], and surprisingly, in vitro studies have shown that concentrations ranging from 0.1 ng/mL to 10 ng/mL can stimulate endothelial, epithelial or keratinocyte’s proliferation, while lower or more excessive amounts actually reduce the rate of healing [13]. Likewise, a clinical trial standardized EGF concentration in CBS eyedrops to 0.15 ng/mL daily, demonstrating a positive effect over the corneal healing process in patients with severe dry eye syndrome (DES) [14].

## 5. UCS Tears: Applications in Ophthalmology

UCS clinical applications in ophthalmology include a wide variety of pathologic ocular surface conditions, such as DES due to Sjögren’s syndrome (or other etiology), persistent epithelial defects (PED), recurrent corneal erosions, neurotrophic keratopathy (NK), graft versus host disease (GVHD), chemical burns causing limbal stem cell deficiency (LCSD), after keratorefractive surgery and in ocular complications associated with SJS, such as ocular surface keratinization and ocular cicatricial pemphigoid [9] (Table 2).

### 5.1. Dry Eye Syndrome (DES)

Yoon et al. used 20% UCS eye drops for 2 months in patients with severe DES showing significant improvement in corneal epithelial staining scores, tear breakup time (TBUT), globet cell density, grade of conjunctival squamous metaplasia and symptoms scores [9]. Another study compared the efficacy between AS and UCS in the treatment of severe DES and demonstrated that symptoms and corneal staining scores were lower in the UCS group. Moreover, in the Sjögren syndrome subpopulation of the same study, goblet cell density increased more in the UCS group compared to the AS group [15]. These data suggest a potent advantage of UCS over AS for the treatment of DES.

### 5.2. Persistent Epithelial Defects (PED)

PEDs can result from lid, tear film or intrinsic epithelial or basement membrane abnormalities, as well as from NK, infections (i.e., herpetic), metabolic disturbances, medications, autoimmune diseases, trauma or chemical burns. A clinical trial in 2015 compared the capacity of UCS vs. AS therapy to promote the healing of resistant conventional medical treatment PEDs. The diameter of the wounds was followed up for 21 days, and the rate of healing was measured as a percentage decrease from baseline measurement at each subsequent checkup. The median percentage decrease in diameter was significantly greater in the UCS group when measured in terms of area and perimeter. Additionally, a larger number of patients achieved complete re-epithelization with UCS compared to AS, suggesting that UCS leads to a more rapid healing process of PEDs compared to AS [16,17,18,19].

### 5.3. Recurrent Corneal Erosions

Recurrent corneal erosion syndrome (RCES) is characterized by repeat episodes of de-epithelization of the cornea that causes ocular pain, tearing, redness and decreased visual acuity. It is caused by DES, mechanical trauma or in the context of corneal dystrophies. Yoon et al. found that by using 20% UCS eyedrops in addition to ATs for a mean period of around 15 months they were able to significantly reduce the recurrence of corneal erosions compared to ATs therapy alone [20,21,22].

### 5.4. Neurotrophic Keratopathy (NK)

NK is a degenerative disease that results from damage to trigeminal corneal innervation leading to impaired sensation and healing of the corneal epithelium. Etiologies include Herpes simplex and Zoster keratitis, mechanical, chemical and surgical injuries and systemic diseases like diabetes mellitus or multiple sclerosis. A prospective, noncomparative case series study applied 20% UCS eye drops to patients with NK, who were not responding to conventional treatment, observing that the epithelial defect healed completely in all patients (100%) within a mean time-frame of just 4 weeks. Visual acuity improved by >2 lines in 60% of cases, and corneal sensitivity also improved after treatment [23,24,25,26].

### 5.5. Graft Versus Host Disease (GVHD)

GVHD is a very common complication in patients receiving allogeneic bone marrow transplants. It typically causes DES, leading to PEDs, punctate keratitis, corneal ulcers and even perforation. A study conducted in South Korea demonstrated that symptom score, TBUT, corneal staining score and sensitivity significantly improved after 6 months of 20% UCS therapy. Compared to AS in the management of DES in patients with GVHD, UCS has the advantage that it is not necessary to collect blood from the patients themselves, whom might be in a poor general condition [27,28,29,30].

### 5.6. Ocular Chemical Burns

After ocular chemical burns, early epithelial healing must occur in order to prevent ulceration, neovascularization and opacification of the cornea. A randomized clinical trial compared UCS therapy versus AS and ATs. The mean time to complete epithelization was reported as 21, 57 and 40 days in UCS, AS and ATs groups, respectively (*p* = 0.02). By day 21 the mean percentage decrease in epithelial defect diameter was found to be 94% with UCS, 53% with AS and 42% with ATs (*p* = 0.01). Long-term complications such as LCSD/Limbal ischemia were assessed after 3 months of therapy, with data showing a mean percentage decrease of 73% with UCS, 36% with AS and 44% with ATs (*p* = 0.008). A greater number of clear corneas were seen in the UCS group compared to the AS or ATs groups (*p* = 0.048). These data are supported by an animal model study comparing the efficacy of 20% UCS, PBS and ATs in the treatment of induced chemical burns. In these experiments, UCS therapy showed improved corneal healing and reduced corneal haze compared to PBS or ATs. Moreover, they demonstrated that IL-1β levels (a molecule that is well known to participate in pyroptosis, an inflammation-induced programmed cell death pathway) [31,32] were significantly reduced in the UCS group compared with the PBS group, suggesting that UCS decreases corneal inflammation more efficiently compared with PBS [33,34].

### 5.7. Keratorefractive Surgeries

Laser epithelial keratomileusis (LASEK) and Laser in situ keratomileusis (LASIK) are procedures that consist of epithelial surface ablation for the correction of refractive errors. The application of 20% UCS eyedrops after surgery significantly reduced mean haze scores and epithelial staining scores, while increases TBUT when compared to conventional treatment [35,36].

## 6. Conclusions

The above studies support the idea that blood-derived products, particularly UCS eye drops, are a safe and efficient option for the treatment of a wide array of ocular surface disease. A major contributing factor is the abundance of essential growth factors and interleukins, which are typically found in the human tear film. However, since each GF and IL selectively participates and regulates different cellular mechanisms involved in corneal healing, the selection of the type of blood-derived product to be used should be decided on the basis of the cellular mechanism causing each pathological case. For this to be achieved, more studies on the specific cellular pathways involved in each ocular surface pathology should be conducted. Future research into the use of blood-derived products will likely focus on the production of tailored eye drops that contain the appropriate GF and IL concentrations and ratios for individualized patient treatment and each ocular surface disease. Moving forward, storage must also be addressed. Currently, the above products must to be refrigerated or frozen to preserve their biological properties and to avoid contamination. This may represent an obstacle for their use in the office setting and for their regulatory compliance. As a consequence, recent efforts have been focused into lyophilizing blood derived products for easier preservation and ambient storage [37,38,39]. However, these studies did not use UCBS but rather AS and plasma rich in growth factors (PRGF). Lastly, artificial reproductions may be a way forward. While the composition of UCBS has been elucidated for some time, there have been no reported attempts to producing an artificial formulation based on these natural products [8,9].

Additional randomized clinical trials, following the same protocol for preparation and utilization in each particular ocular pathology, are needed to provide clearer evidence, improve the quality of final products and provide a better understanding and widespread application of these therapies in daily ophthalmologic clinical practice [3,5,8].

## Figures and Tables

**Table 1:** Comparison of growth factor concentrations in UCS and PBS.

	Elevated Concentration in UCS	Elevated Concentration in PBS
	EGF	IGF-1
	TGF-α	IGF-2
	TGF-β2	TGF-β1
	FGF	Vitamin A
	PDGF	
Growth factor	VEGF	
	NGF	
	IL-1B	
	IL-4	
	IL-6	
	IL-10	
	IL-13	

UCS: Umbilical cord serum; PBS: Peripheral blood serum; GFs: Growth factors; EGF: epidermal growth factor; TGF: transforming growth factor; FGF: fibroblast growth factor; PDGF: platelet-derived growth factor; VEGF: vascular endothelial growth factor; NGF: nerve growth factor; IL: Interleukin; IGF: insulin-like growth factor. The majority of the growth factors, cytokines and Interleukin’s concentrations were found to be greater in UCS compared to PBS, while only the levels of IGF-1, IGF-2, TGF-β1 and Vitamin A were significantly greater in PBS compared to UCS [8].

**Table 2:** Umbilical cord serum tears applications in ophthalmology.

Study	Condition	Sample Size	Dilution	Dosage	Duration	Concomitant	Results (Statistically Significant)
Yoon et al. [9]	DES	31	20%	6–10/day	2 months	ATs	Improvement in symptom score, TBUT and CS, grade of squamous metaplasia and globet cell density.
Yoon et al. [15]	DES	48	20%	6–10/day	2 months	PFATs	UCS tears were superior than AS tears: Improvement in symptoms, CS, and in the Sjögren subpopulation, globet cell density increased more with UCS than with AS.
Valpayee et al. [18]	PED	59	20%	-	21 days	None (Prior 1-week washout period)	Decrease in diameter was greater with UCS compared to AS tears. More patients using UCS achieved complete re-epithelization.
Yoon et al. [22]	RCE	35	20%	4–6/day	15 months	ATs	Treatment with UCS eyedrops in addition to ATs significantly reduced the recurrence of corneal erosions compared to ATs therapy alone.
Yoon et al. [26]	NK	28	20%	6–10/day	4 weeks	PFATs	Epithelial defects healed completely in all patients within 4 weeks. VA improved by >2 lines in 60%. Corneal sensitivity also improved after treatment.
Yoon et al. [30]	GVHD	12	20%	6–10/day	6 months	ATs	Symptom score, TBUT, CS, corneal sensitivity improved after 6 months of treatment with UCS.
Sharma et at. [34]	OCB	32	20%	10/day	3 months	0.3% Ofloxacin, Prednisolone acetate 1%, homatropine hydrobromide 2%, Ascorbate 10%, PFATs, Antiglaucoma drops if required.	Complete re-epithelization was achieved first in the UCS group compared to the AS and ATs group. Long term complications (LSCD) were less frequent in the USC group.
Oh et al. [33]	OCB	24 mice (Animal model)	20%	4/day	7 days	Topical levofloxacin.	UCS therapy showed improved corneal healing and reduced corneal haze compared to PBS or ATs.
Yoon et al. [36]	After LASEK	60	20%	4–6/day	12 weeks	Conventional treatment: Antibiotics, steroids and ATs.	UCS therapy after surgery reduced mean haze scores and CS scores, while it increased BUT when compared to conventional treatment only.

UCS: Umbilical cord serum. AS: Autologous serum. PBS: Peripheral blood serum. ATs: Artificial tears. PFATs: Preservative-free ATs. TBUT: Tear breakup time. CS: Corneal staining. VA: Visual acuity. DES: Dry eye syndrome. PED: Persistent epithelial defect. RCE: Recurrent epithelial erosions. NK: Neurotrophic keratopathy. GVHD: Graft versus host disease. OCB: Ocular chemical burn. LASEK: Laser epithelial keratomileusis. Multiple clinical trials have demonstrated the efficacy of UCS in the treatment of several ocular surface conditions, demonstrating their superiority over standard or conventional therapies. A subset of trials also compared the healing potential of UCS to other blood derived products, such as AS tears, finding more encouraging and consistent results with UCS products.
